# Deep learning–based time-of-flight (ToF) image enhancement of non-ToF PET scans

**DOI:** 10.1007/s00259-022-05824-7

**Published:** 2022-05-04

**Authors:** Abolfazl Mehranian, Scott D. Wollenweber, Matthew D. Walker, Kevin M. Bradley, Patrick A. Fielding, Martin Huellner, Fotis Kotasidis, Kuan-Hao Su, Robert Johnsen, Floris P. Jansen, Daniel R. McGowan

**Affiliations:** 1 GE Healthcare, Big Data Institute, University of Oxford, Oxford, UK; 2 GE Healthcare, Waukesha, WI USA; 3 Department of Medical Physics and Clinical Engineering, Oxford University Hospitals NHS FT, Oxford, UK; 4 Wales Research and Diagnostic PET Imaging Centre, University Hospital of Wales, Cardiff, UK; 5 Department of Radiology, University Hospital of Wales, Cardiff, UK; 6 Zurich University Hospital, Zurich, Switzerland; 7 GE Healthcare, Zurich, Switzerland; 8 Department of Oncology, University of Oxford, Oxford, UK

**Keywords:** Deep neural networks, Time of flight, PET, Image quality

## Abstract

**Purpose:**

To improve the quantitative accuracy and diagnostic confidence of PET images reconstructed without time-of-flight (ToF) using deep learning models trained for ToF image enhancement (DL-ToF).

**Methods:**

A total of 273 [^18^F]-FDG PET scans were used, including data from 6 centres equipped with GE Discovery MI ToF scanners. PET data were reconstructed using the block-sequential-regularised-expectation–maximisation (BSREM) algorithm with and without ToF. The images were then split into training (*n* = 208), validation (*n* = 15), and testing (*n* = 50) sets. Three DL-ToF models were trained to transform non-ToF BSREM images to their target ToF images with different levels of DL-ToF strength (low, medium, high). The models were objectively evaluated using the testing set based on standardised uptake value (SUV) in 139 identified lesions, and in normal regions of liver and lungs. Three radiologists subjectively rated the models using testing sets based on lesion detectability, diagnostic confidence, and image noise/quality.

**Results:**

The non-ToF, DL-ToF low, medium, and high methods resulted in − 28 ± 18, − 28 ± 19, − 8 ± 22, and 1.7 ± 24% differences (mean; SD) in the SUV_max_ for the lesions in testing set, compared to ToF-BSREM image. In background lung VOIs, the SUV_mean_ differences were 7 ± 15, 0.6 ± 12, 1 ± 13, and 1 ± 11% respectively. In normal liver, SUV_mean_ differences were 4 ± 5, 0.7 ± 4, 0.8 ± 4, and 0.1 ± 4%. Visual inspection showed that our DL-ToF improved feature sharpness and convergence towards ToF reconstruction. Blinded clinical readings of testing sets for diagnostic confidence (scale 0–5) showed that non-ToF, DL-ToF low, medium, and high, and ToF images scored 3.0, 3.0, 4.1, 3.8, and 3.5 respectively. For this set of images, DL-ToF medium therefore scored highest for diagnostic confidence.

**Conclusion:**

Deep learning–based image enhancement models may provide converged ToF-equivalent image quality without ToF reconstruction. In clinical scoring DL-ToF-enhanced non-ToF images (medium and high) on average scored as high as, or higher than, ToF images. The model is generalisable and hence, could be applied to non-ToF images from BGO-based PET/CT scanners.

**Supplementary Information:**

The online version contains supplementary material available at 10.1007/s00259-022-05824-7.

## Introduction

Time-of-flight (ToF) positron emission tomography (PET) is a detector technology that measures the arrival times of the annihilation photons with an uncertainty governed by the coincidence timing resolution (CTR) of the scanner [1]. The first generation of ToF PET scanners were equipped with either caesium or barium fluoride scintillators coupled with photomultiplier tubes, providing CTR of 400–600 ps. However, neither their sensitivity nor spatial resolution could compete with that of non-ToF bismuth germanate (BGO)–based scanners. With the advent of lutetium (Lu)-based scintillators with better sensitivity and spatial resolution, conventional photomultiplier tube–based ToF PET scanners became commercially available around 2006 with a CTR in the range of 450–600 ps. Since then, advancements in silicon photomultiplier detectors have led to the next generation of ToF scanners with CTR of 214–380 ps [2]. Compared to Lu-based scintillators, BGO has a higher stopping power, therefore higher sensitivity for a given crystal size, at the expense of poor timing resolution.

Using ToF capability, the location of emission points along each line of response (LOR) is estimated and utilised during PET image reconstruction to update image voxels only along each segment of response, defined by ToF resolution, instead of the whole LOR. Consequently, the cross-dependencies between image voxels are reduced, which results in (i) reduced noise propagation with fast and space-invariant convergence, which in turn improves the detectability of lesions [3, 4], and (ii) reduced sensitivity to errors in normalization, attenuation correction, and scatter correction [5, 6]. As the CTR is improved, cross-dependencies between image voxels are reduced leading to further ToF benefits.

ToF technology together with advanced image reconstruction algorithms (such as ordered subsets expectation maximisation — OSEM, or block sequential regularised expectation maximisation — BSREM [7]) have led to improved diagnostic confidence and lesion detectability [8]. With the recent advancements in artificial intelligence, deep learning (DL) techniques have found promising applications in PET imaging from photon detection to image reconstruction [9–11]. Recently, deep convolutional neural networks have been extensively used to reduce PET acquisition time or radiotracer dose by reducing image noise [12–14] or reconstruction time with improved image convergence [15]. For ToF technology, DL has been used for data-driven time-of-flight estimation which can lead to about 20% improvement in CTR [16].

Given that the benefits of ToF technology are directly translated into image space and a number of current clinical PET scanners are non-ToF (i.e. BGO-based), there is a desire to improve the diagnostic value of non-ToF scanners compared to ToF scanners [17, 18]. In this study, we aim to leverage deep learning to enable ToF benefits for PET images reconstructed without ToF information. To the best of our knowledge, the proposed deep learning for ToF image enhancement (DL-ToF) is the first-ever attempt to transform non-ToF PET images to ToF-like images. Although related, this challenge differs substantially from the aforementioned uses of DL for noise-reduction due to the variety of ways in which ToF information influences the image appearance. The neural network is not required to add time of flight information to the PET coincidence data, but it is required to learn how ToF information alters many image characteristics, and then to replicate these changes when supplied with non-TOF input images. Three DL-ToF models with different levels of contrast-enhancement-to-noise trade off (low: L, medium: M and high: H) were trained in supervised learning sessions for transforming non-ToF BSREM images, each reconstructed with a range of regularization parameters (beta), to ToF BSREM images, reconstructed with specific regularization values to reflect the intended level of contrast-to-noise. The performance of the three models was quantitatively and qualitatively evaluated using ToF and non-ToF PET scans for [^18^F]-FDG oncology exams.

## Materials and methods

### Data acquisition and processing

The PET list-mode data and CT-based attenuation correction (CTAC) images of a total of 273 whole-body oncology [^18^F]-FDG PET exams were retrospectively collected, as summarised in Supp. Materials Table 1, from six clinical sites equipped with Discovery MI (DMI) and D710 ToF PET/CT scanners. Using training datasets from various clinical sites improves the generalisability of DL-ToF models to account for the fact that each site uses different acquisition protocols and reconstruction parameters. The DMI’s PET subsystem has ToF resolution of 385 ps and different sensitivity depending on the number of detector rings. The DMI system can be configured with 3, 4, or 5 rings of detectors, for axial FOV of 15, 20 or 25 cm. The D710 has a ToF resolution of 550 ps and an axial FOV of 15.7 cm. The use of different scanners and imaging protocols at different sites led to a range of injected [^18^F]-FDG activity (mean ± SD: 348 ± 118 MBq) and scan duration (161 ± 46 s/bed). Moreover, there were variations in patient size (body mass index, BMI, 27.3 ± 6.0 kg/m^2^). The [^18^F]-FDG uptake time varied between sites: 82 ± 26 min. For each subject, a whole-body CT scan protocol was performed for PET attenuation correction using 100–120 kVp.

The 273 DMI/D710 exams were divided into training (*n* = 208), validation (*n* = 15), and testing (*n* = 50) sets. The validation and testing DMI exams were chosen by two nuclear medicine experts for pathologically interesting cases with small lesions. Each dataset was reconstructed using the BSREM algorithm into 4 image series, one ToF (target), and three non-ToF images (input) with different beta values. Supp. Materials Table 1 summarises the beta values chosen for each DL-ToF model, clinical site, and target-input pair. The beta value of ToF BSREM images were experimentally adjusted per site in order to achieve the same low noise level, based on visual inspection, across data from all sites. Each image was reconstructed with a matrix size of 256 × 256 and field-of-view of 700 mm (*x*–*y* pixel size: 2.73 mm, slice thickness: 2.79 mm). The whole-body image volumes used for training and validation were axially divided into equally spaced contiguous 3D sub-volumes, each of 100 slices (28 cm).

### Model training

A 3D residual U-Net network [19] was developed and implemented in PyTorch 1.6 (www.pytorch.org) shown in Supp. Figure 1. DL-ToF networks were trained in a supervised session in which their predicted ToF BSREM images were compared to target ToF BSREM ones based on a mean squared error (MSE) loss function. Supp. Materials Table 2 summarises the network and training hyperparameters that were optimised experimentally. The ADAM algorithm [20] was used to update the networks’ trainable parameters for a maximum of 100 epochs on a workstation with two RTX6000 GPUs. The validation set was used to monitor the network’s generalisation error to avoid over-fitting. The epoch at which the model had the lowest validation loss was chosen as a stopping point.

### Evaluation

The performance of our trained DL models was quantitatively evaluated using the testing sets based on standardised uptake values (SUVs) including SUV_max_ (maximum voxel intensity) in lesions, SUV_mean_ (mean intensity of voxels) in normal liver and lungs and the noise in the liver using volumes of interest (VOIs) selected per subject. For each subject, 5 VOIs of size 7 × 7 × 7 voxels (~ 7 mL) were defined in the lungs, and 5 similar VOIs in liver. Noise in liver was calculated as standard deviation of the five VOI mean values. For each subject, up to 5 small lesions were visually identified and segmented using an adaptive thresholding method (42% of maximum minus minimum SUV in a 7 × 7 × 7 bounding box). For the evaluation of DL-ToF models, the beta value of both non-ToF and ToF BSREM images were set to 350. The difference in SUV values (compared to the target ToF BSREM SUVs), scatter plots, and Bland–Altman plots were generated. The statistical significance of differences in SUVs was evaluated using the Wilcoxon signed rank test. Additionally, root-mean-square error (RMSE) between reference ToF images (*x*) and other images (*y*) over the whole-body (WB) was calculated by $RMSE=\sqrt{\frac{1}{N}{\sum }_{i}^{N}{\left({x}_{i}-{y}_{i}\right)}^{2}}$, where *N* is the total number of voxels in the body. Also, WB SUV_mean_ was calculated by averaging the SUV values in the body.

Three radiologists, (KMB, PAF, and MH), blinded to image reconstruction, independently rated all 50 testing sets. Each patient had 5 image series (Non-ToF and ToF BSREM, DL-ToF low (L), medium (M) and high (H)); these were assessed, with corresponding CT, based on Likert scores considering three image features (lesion detectability, diagnostic confidence, and image noise/quality). The Likert scale used was 0 (non-diagnostic), 1 (poor,), 2 (satisfactory), 3 (good), 4 (very good), and 5 (excellent) with image noise metrics scored on the same 0–5 scale as described previously [21]. In addition, the 5 series were ranked in order of preference from 1 (best) to 5 (worst) for each imaging feature category. Using SPSS v27, the interclass correlation coefficient (ICC) was calculated between the radiologists’ image scores to assess the level to which the average reader scores are generalisable to a wider population of readers [22]. Post hoc pairwise testing against both non-ToF and ToF BSREM images was then performed using Dunn’s method, with Bonferroni correction applied to the reported *p*-values.

### Application to a BGO-PET camera

Ten exams acquired on a GE Discovery IQ (DIQ) scanner were used to illustrate the generalisability of the trained models for a non-ToF BGO-based scanner. This final step of running DL-ToF models on data from a non-TOF, BGO PET scanner had the goal of demonstrating the potential of the DL approach. However, with no ground truth or target solution to compare against, we limited this part of the study to a small number of subjects, and the image analysis to a visual verification that the images were free of obvious artefacts and showed visual changes in accordance with expectations for the three DL models.

## Results

**Fig. 1:**
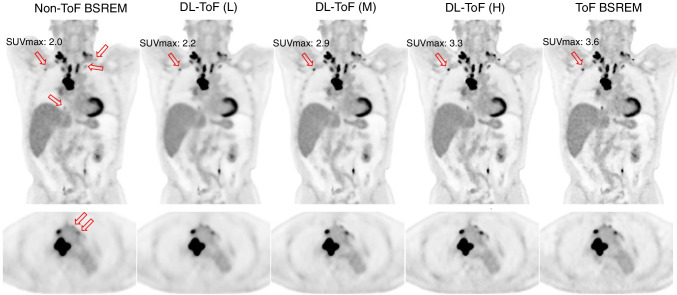
DL-ToF enhancement of a representative test subject with a BMI of 26.4 kg/m^2^ with an injected activity of 515 MBq scanned on GE Discovery MI (5-ring) PET/CT scanner (slice thickness 2.8 mm). Arrows point to lesions with lower detectability in non-ToF BSREM as well as the SUV_max_ of an example lesion (SUV_max_ values of all investigated lesions are summarised in Table 1 and shown in Fig. 3). Display window: 0–5 SUV

**Fig. 2:**
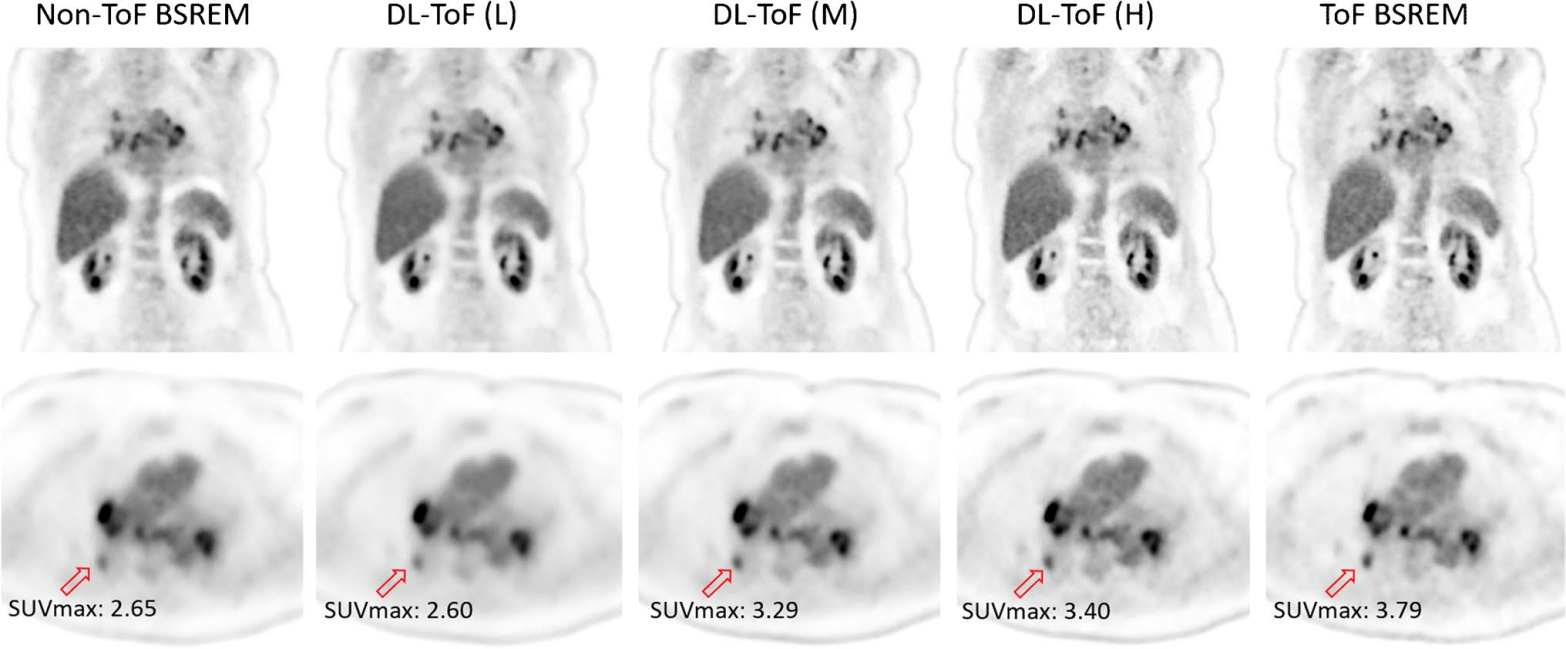
DL-ToF enhancement of a representative test subject with a BMI of 31.6 kg/m^2^ with an injected activity of 514 MBq scanned on GE Discovery MI (5-ring) PET/CT scanner (slice thickness 2.8 mm). Arrows point to an example lesion with SUV_max_ shown (SUV_max_ values of all investigated lesions are summarised in Table 1 and shown in Fig. 3). Display window: 0–5 SUV

Figures 1 and 2 compare the performance of our three DL-ToF models in comparison with the input non-ToF BSREM (beta = 350) and target ToF BSREM (beta = 350) images for two representative, example patients with different BMI scanned on a Discovery MI PET/CT scanner (further examples are shown in Supp. Figures 2–5). As shown by the arrows, the patients have multiple small lesions in different areas (neck, mediastinum, breast, and vertebrae) which have a lower contrast in the non-ToF image. The DL-ToF models improve the detectability and contrast of the lesions towards their target ToF images, with DL-ToF(H) providing the closest match visually. Since the models were trained to provide different levels of smoothness, the liver noise as well as lesion contrast is different among these three models. As shown in Fig. 1, the DL-ToF models improve the overall image quality and feature sharpness of the non-ToF PET images.

**Table 1:** Quantitative performance of the DL-ToF models evaluated on 50 test exams, expressed as a percentage difference with ToF BSREM (taken as ground truth), for lesion SUV_max_, lung SUV_mean_, liver SUV_mean_, and noise in liver (the standard deviation of noise averaged over all exams) for each type of reconstruction. *P*-values (parentheses) show significance of difference from ToF BSREM

	Lesion SUV_max_ (%)	Lung SUV_mean_ (%)	Liver SUV_mean_ (%)	Liver noise (SUV)
Non-ToF BSREM	− 28.6 ± 18.3 (< 0.0001)	7.7 ± 15.0 (< 0.0001)	4.3 ± 5.6 (< 0.0001)	0.16
DL-ToF(L)	− 28.7 ± 19.0 (< 0.0001)	0.6 ± 12.1 (0.179)	0.7 ± 4.6 (0.067)	0.10
DL-ToF(M)	− 8.0 ± 22.5 (< 0.0001)	1.3 ± 13.0 (0.083)	0.8 ± 4.4 (0.016)	0.13
DL-ToF(H)	1.7 ± 23.9 (0.57)	1.4 ± 11.5 (0.50)	0.1 ± 4.5 (0.86)	0.19
ToF-BSREM	–	–	–	0.19

**Fig. 3:**
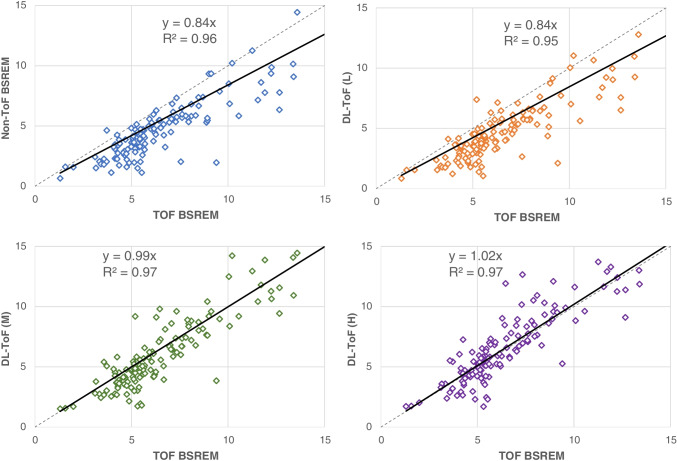
Scatter plots of lesion SUV_max_ for non-ToF BSREM and different DL-ToF models compared to ToF BSREM images. The grey dashed line is an identity line. Each dot corresponds to a lesion

Table 1 shows the quantitative performance of non-ToF BSREM and DL-ToF methods on the DMI’s testing set (*n* = 50) for SUV_max_ of the 139 identified lesions, and SUV_mean_ in normal lungs and liver. The percentage difference from the target ToF BSREM method is provided (mean ± standard deviation), along with the result of the tests of statistical significance. As seen, DL-ToF methods reduce the lesion’s SUV_max_ difference from − 28.6 to 1.7%, depending on their level of smoothness. In this test dataset, the difference of 1.7% between DL-ToF(H) and ToF BSREM was not statistically significant. In the lungs, DL-ToF models reduce the SUV_mean_ differences from 7.7% to less than 2%. These results demonstrate that DL-ToF models make lesions hotter and lungs colder; in other words, they improve the accuracy of the non-ToF BSREM images. Assessment of the noise in the liver, via the average of the liver standard deviations from 50 testing datasets, shows that the DL-ToF models provide different level of smoothness, and all provide some level of noise reduction. Supp. Materials Table 3 shows the RMSE and SUV_mean_ over whole-body and lesion/lung/liver ROIs for the test cases.

Figure 3 shows scatter plots of lesion SUV_max_ for non-ToF BSREM and DL-ToF images compared to reference ToF BSREM images including slope and R-square of regression lines. As could be expected, the non-ToF BSREM method shows a lower lesion SUV_max_ and hence a less steep slope of the fitted line compared to ToF BSREM method. As the strength of DL-ToF is increased, the slope of fitted line for DL-ToF methods gets closer to identity: this indicates contrast convergence enhancement of the input non-ToF images. As shown, DL-ToF(H) increases the slope from 0.84 to 1.02 and increases the coefficient of determination (0.96 to 0.97).

**Fig. 4:**
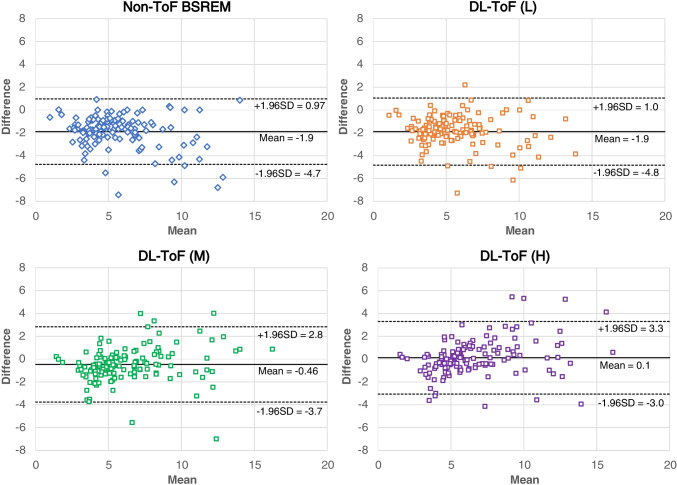
Bland–Altman plots comparing the concordance of lesion SUV_max_ between ToF BSREM and other reconstruction methods. Each dot corresponds to a lesion

Figure 4 shows Bland–Altman plots comparing the concordance of lesion SUV_max_ between target ToF BSREM and other methods. Consistent with the other quantification measures, the plots show a systematic difference in SUV_max_ which is reduced by DL-ToF methods.

Table 2 and Supp. Materials Table 4 show the scores and ranking results for different reconstruction methods for 50 testing exams from three independent readers. Table 2 also provides *p*-values for the scores, using pairwise comparisons with respect to ToF BSREM methods. Supp. Materials Table 5 shows *p*-values with respect to non-ToF BSREM. The lesion detectability results show that DL-ToF(H) significantly improves lesion detectability in the images so much that their *p*-values become lower than 0.001. In terms of diagnostic confidence, DL-ToF(M) achieves the best score whereas for image noise/quality DL-ToF(L) scores the best. These results highlight that the strength of DL-ToF can be chosen to provide a balance between lesion detection and noise reduction, according to the preference of the image reader. Results in Supp. Materials Table 4 also show DL-ToF(H) achieves the best rank for lesion detectability, whereas DL-ToF (M) has the best rank for diagnostic confidence (i.e., better than ToF BSREM) and DL-ToF(L) achieves the best rank in terms of noise and image quality.

**Table 2:** Clinical image quality scoring from three readers of 50 test whole-body scans based on different criteria, mean ± standard deviation. 0 is non-diagnostic; 5 is excellent. Bold indicates the best (highest) score for each metric. The intraclass correlation coefficient (ICC) is also provided for each metric (95% confidence interval) to show reader agreement. *P*-values (in parentheses) are given with respect to ToF BSREM (with *p*-values with respect to non-ToF BSREM shown in Suppl. Table 5)

Scores	Diagnostic confidence	Lesion detectability	Image noise/quality
Non-ToF BSREM	3.03 ± 0.40 (< 0.001)	3.03 ± 0.43 (< 0.001)	3.36 ± 0.40 (1.000)
DL-ToF(L)	2.98 ± 0.34 (< 0.001)	2.88 ± 0.35 (< 0.001)	**4.52 ± 0.27** (< 0.001)
DL-ToF(M)	**4.07 ± 0.47** (< 0.001)	3.99 ± 0.48 (1.000)	4.09 ± 0.34 (< 0.001)
DL-ToF(H)	3.83 ± 0.38 (0.11)	**4.18 ± 0.39** (1.000)	3.39 ± 0.40 (0.96)
ToF BSREM	3.53 ± 0.53	4.08 ± 0.54	3.08 ± 0.55
ICC	0.67 (0.60, 0.74)	0.68 (0.61, 0.74)	0.58 (0.48, 0.66)

**Fig. 5:**
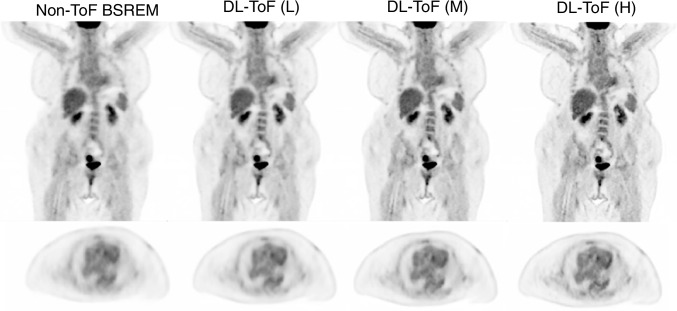
DL-ToF enhancement of a representative test subject with a BMI of 53.8 kg/m^2^ and weight of 93.9 kg with an injected activity of 344 MBq scanned on a GE Discovery IQ non-ToF PET/CT scanner (slice thickness 3.8 mm). Display window: 0–5 SUV

**Fig. 6:**
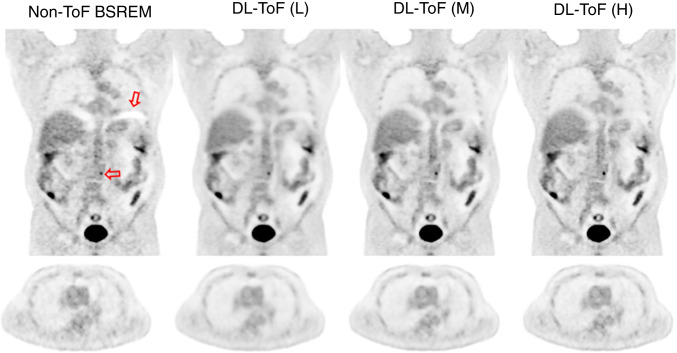
DL-ToF enhancement of a representative test subject with a BMI of 26.0 kg/m^2^ and weight of 84.8 kg with an injected activity of 160 MBq scanned on a GE Discovery IQ non-ToF PET/CT scanner (slice thickness 3.8 mm). The arrow shows attenuation artefacts and a small lesion in vertebra. Display window: 0–5 SUV

The application of the technique to data from a BGO non-ToF DIQ PET scanner (on which the algorithm had not been trained) provided images which, visually, met our expectations. This is shown in Figs. 5 and 6, which although from different patients, can be compared to Figs. 1 and 2. Eight further examples are presented in Supplementary Figs. 6–9. In these cases, the models showed similar image enhancement as was achieved with non-ToF DMI data. In Fig. 6, the patient presents attenuation correction artefacts near to the diaphragm, which are often reduced by ToF reconstruction [6]; in this instance, all DL-ToF models show reduction of the artefacts.

## Discussion

In this study, the feasibility of utilising deep learning for enhancing the ToF features in PET images reconstructed without ToF capability was explored with the intended application of improving lesion detectability and diagnostic confidence for [^18^F]-FDG PET scans acquired in non-ToF PET scanners. Three DL-ToF models were trained with different levels of smoothness or ToF strength to demonstrate the flexibility of the proposed DL solution to meet radiologists’ preferences.

Our models were trained to transform non-ToF PET images reconstructed by the BSREM algorithm to their corresponding ToF BSREM images as faithfully as possible. The BSREM algorithm was chosen over OSEM as it provides a higher convergence and lower noise, therefore giving more accurate quantification [7, 21]. The DL-ToF(H) algorithm that was developed, when applied on non-ToF BSREM (beta = 350) input data, achieves quantification errors less than 5% compared to the target ToF BSREM. On the other hand, the results show that all DL-ToF models provide some level of noise reduction, which translates to improved contrast-to-noise ratio (CNR), indicating improved feature sharpness and lesion detectability.

As shown in Fig. 3, in our primary testing set of 50 exams, the identified lesions are mostly clustered around an SUV_max_ of 5 in the ToF BSREM images (median = 5.6). These lesions, as well as those of lower SUV_max_, are often diagnostically important and affected by ToF reconstruction method. The scatter plots show that as one moves from DL-ToF(L) to DL-ToF(H) the set of lesion SUVs are increased toward their target ToF SUVs.

The clinical reading results showed that DL-ToF models present favourable performance. For instance, in Table 2, DL-ToF(H), which has the least smooth DL-ToF model or highest ToF strength, achieves on average 3.83, 4.18, and 3.39 scores for the key metrics of diagnostic confidence, lesion detectability, and image noise/quality compared to reference ToF images with the corresponding scores of 3.53, 4.08, and 3.08, respectively. In our test set, the best score for lesion detectability was from DLT(H) with the best score for image noise/quality from DLT(L) and diagnostic confidence from DLT(M). Overall, in terms of diagnostic confidence, the DL-ToF(M) model provides a better trade-off in our test set as a lower noise and improved detectability are desirable features for an image reconstruction or enhancement technique. It is the balance of good performance regarding lesion detectability and image noise that leads to best diagnostic confidence as shown by the blinded clinician scores and ranking. In some implementations of PET image reconstruction, including that used here, the use of ToF information can lead to faster convergence and hence a noisier image as compared to non-TOF for the same number of iterations. It is possible to smooth ToF images with a Gaussian filter or in the case of BSREM to use a larger beta value but this may come at the cost of a reduction in lesion detectability [7].

This study utilised a U-Net model, as an encoder-decoder CNN, that was trained using a diverse set of DMI datasets. The quality and diversity of the training set is one of the key factors in the performance and generalisability of a CNN model. To exemplify this generalisability, the models were also applied to ten patients scanned on a BGO non-ToF scanner (GE Healthcare Discovery IQ), as shown in Figs. 5 and 6 and Supplementary Figs. 6–9. These examples suggest that the models may work on data from scanners that were not part of the training dataset; examining this in more detail will be the subject of a follow-on study.

This study has a number of limitations. Our testing sets do not include randomly selected exams (i.e. combination of normal/abnormal) but rather patients with lesions that showed low activity or which were completely missed in non-ToF BSREM images. Therefore, our results might be biased to highlight the gap between ToF and non-ToF reconstructions. However, our results with another testing set used during model validation (not shown in this study) demonstrated that our DL-ToF models show the greatest enhancement for patients with the highest BMI; this result is in line with the expected behaviour of ToF reconstruction. Another limitation could be that the readers were shown all 5 sets of images (blinded) of a subject at the same time. This might bias the scoring of the images, although was considered advantageous, in order to facilitate the detection of false positive or missing lesions by comparing images all at once and furthermore is required in order to produce a rank ordering. Further, DL-ToF models were not compared to any other ToF image enhancement technique given the novelty of our methodology and they were not tested for non-FDG tracers. Therefore, this work opens new research topics for future studies. Future work should include further clinical evaluation using a cohort of FDG exams with the possibility of getting the clinical feedback into the training cycle of our models.

## Conclusion

This study developed three deep convolutional neural networks for ToF-like enhancement of PET images acquired in non-ToF PET/CT scanners. Our results demonstrate that the proposed networks improve the feature quantification (lesions, liver and lungs), overall image sharpness (as seen with ToF, e.g. organ delineation, ribs, vertebrae), and overall diagnostic value (particularly in terms of lesion detectability and diagnostic confidence). Depending on the model ToF strength, DL-ToF(L) showed more noise reduction, whereas DL-ToF(H) had the greatest improvement in lesion detection. DL-ToF(M) presented a balanced performance and best diagnostic confidence. We conclude that deep learning–enhanced image reconstruction can markedly improve non-ToF PET images towards their corresponding ToF images.

## Supplementary Information

Below is the link to the electronic supplementary material.

Supplementary file1 (DOCX 10102 KB)

## Data Availability

Data is available under reasonable request to the corresponding author.

## Code availability

The code used in the preparation of this paper is not available for public distribution.
