# Supplementary material for: Deep learning–based time-of-flight (ToF) image enhancement of non-ToF PET scans
Source: Eur J Nucl Med Mol Imaging. 2022 May 4;49(11):3740–9. doi: 10.1007/s00259-022-05824-7 (PMC9399038; doi:10.1007/s00259-022-05824-7)
Supplement: Supplementary file 1 — Supplementary file1 (DOCX 10102 KB) [file 259_2022_5824_MOESM1_ESM.docx]

**Supplementary Table 1** Scanner and reconstruction parameters used in this study

| **Site** | **Scanner** | **# exams** | | | **ToF BSREM beta** | | | **Non-ToF BSREM beta** |
| --- | --- | --- | --- | --- | --- | --- | --- | --- |
|  |  | Training | Validation | Testing | Low | Medium | High |  |
| A | MI 4R | 57 | 0 | 2 | 1000 | 500 | 375 | 200, 350, 500, 750 |
| B | MI 5R | 80 | 10 | 20 | 850 | 350 | 250 | 200, 350, 500, 750 |
| C | MI 5R | 49 | 1 | 13 | 1050 | 550 | 400 | 200, 350, 500, 750 |
| D | MI (3R, 4R) | 22 | 3 | 1 | 1050 | 550 | 400 | 200, 350, 500, 750 |
| E | MI 4R | 0 | 1 | 0 | 850 | 350 | 250 | 200, 350, 500, 750 |
| F | D710 | 0 | 0 | 14 | --- | 400 | --- | 350 |
| Average | --- | --- | | | 960 | 450 | 335 | --- |

**Supplementary Table 2.** Model specifications and training parameters used in this study

| **Model** | **No. trainable parameters** | **No. kernels in 1^st^ layer** | **No. max pooling layers** | **Kernel size** | **Batch size** | **Learning rate** | **No. epochs** | **Optimiser** | **Loss function** |
| --- | --- | --- | --- | --- | --- | --- | --- | --- | --- |
| DL-ToF(L, M, H) | 40,158,593 | 64 | 4 | 3×3×3 | 8 | 0.005 | 100 | Adam | MSE |

**Supplementary Table 3**. Root mean square error (RMSE) and SUV_mean_ over whole-body (WB) and different ROIs for 50 testing. RMSE are with respect to TOF BSREM.

| **Model** | **RMSE (WB)** | **RMSE**  **(lesions)** | **RMSE**  **(liver)** | **RMSE**  **(lung)** | **SUVmean**  **(WB)** | **SUVmean**  **(lesions)** | **SUVmean**  **(liver)** | **SUVmean**  **(lung)** |
| --- | --- | --- | --- | --- | --- | --- | --- | --- |
| Non-ToF BSREM | 0.171 | 1.51 | 0.18 | 0.1 | 0.69 ± 0.06 | 3.60 ± 6.40 | 2.5 ± 0.5 | 0.52 ± 0.24 |
| DL-ToF (L) | 0.174 | 2.43 | 0.12 | 0.07 | 0.71 ± 0.05 | 3.48 ± 4.85 | 2.5 ± 0.4 | 0.48 ± 0.21 |
| DL-ToF (M) | 0.155 | 1.13 | 0.11 | 0.07 | 0.70 ± 0.05 | 4.55 ± 7.28 | 2.5 ± 0.4 | 0.49 ± 0.21 |
| DL-ToF (H) | 0.163 | 0.97 | 0.12 | 0.06 | 0.70 ± 0.05 | 4.90 ± 6.92 | 2.4 ± 0.4 | 0.49 ± 0.21 |
| ToF BSREM | ---- | ---- | ---- | --- | 0.69 ± 0.05 | 4.78 ± 6.60 | 2.4 ± 0.4 | 0.48 ± 0.2 |

**Supplementary Table 4**.

Clinical image quality ranking from three readers of 50 testing scans based on different criteria, mean ± standard deviation. 1 is best and 5 is worst. Bold indicates the best (lowest) rank for each metric.

| **Ranks** | **Diagnostic**  **confidence** | **Lesion**  **detectability** | **Image noise/quality** |
| --- | --- | --- | --- |
| Non-ToF BSREM | 3.8 ± 0.46 | 3.8 ± 0.38 | 3.2 ± 0.11 |
| DL-ToF(L) | 4.2 ± 0.32 | 4.4 ± 0.30 | **2.1 ± 1.41** |
| DL-ToF(M) | **1.9 ± 0.42** | 2.3 ± 0.28 | 2.2 ± 0.12 |
| DL-ToF(H) | 2.1 ± 0.07 | **1.9 ± 0.10** | 3.2 ± 0.79 |
| ToF BSREM | 2.8 ± 0.17 | 2.3 ± 0.34 | 3.9 ± 0.68 |

**Supplementary Table 5**.

Clinical image quality scoring from three readers of 50 testing exams scans based on different criteria, mean ± standard deviation. 0 is non-diagnostic, 5 is excellent. Bold indicates the best (highest) score for each metric. The Intraclass Correlation Coefficient (ICC) is also provided for each metric [95% confidence interval] to show reader agreement. *P*-values (in parenthesis) are given with respect to non-ToF BSREM (Table 2 shows these with respect to ToF BSREM).

| **Scores** | **Diagnostic**  **confidence** | **Lesion**  **detectability** | **Image noise/quality** |
| --- | --- | --- | --- |
| Non-ToF BSREM | 3.03 ± 0.40 | 3.03 ± 0.43 | 3.36 ± 0.40 |
| DL-ToF(L) | 2.98 ± 0.34  (0.39) | 2.88 ± 0.35  (0.18) | **4.52 ± 0.27**  (<0.001) |
| DL-ToF(M) | **4.07 ± 0.47**  (<0.001) | 3.99 ± 0.48  (<0.001) | 4.09 ± 0.34  (<0.001) |
| DL-ToF(H) | 3.83 ± 0.38  (<0.001) | **4.18 ± 0.39**  (<0.001) | 3.39 ± 0.40  (1.000) |
| ToF BSREM | 3.53 ± 0.53  (<0.001) | 4.08 ± 0.54  (<0.001) | 3.08 ± 0.55  (1.000) |
| ICC | 0.67 [0.60, 0.74] | 0.68 [0.61, 0.74] | 0.58 [0.48, 0.66] |


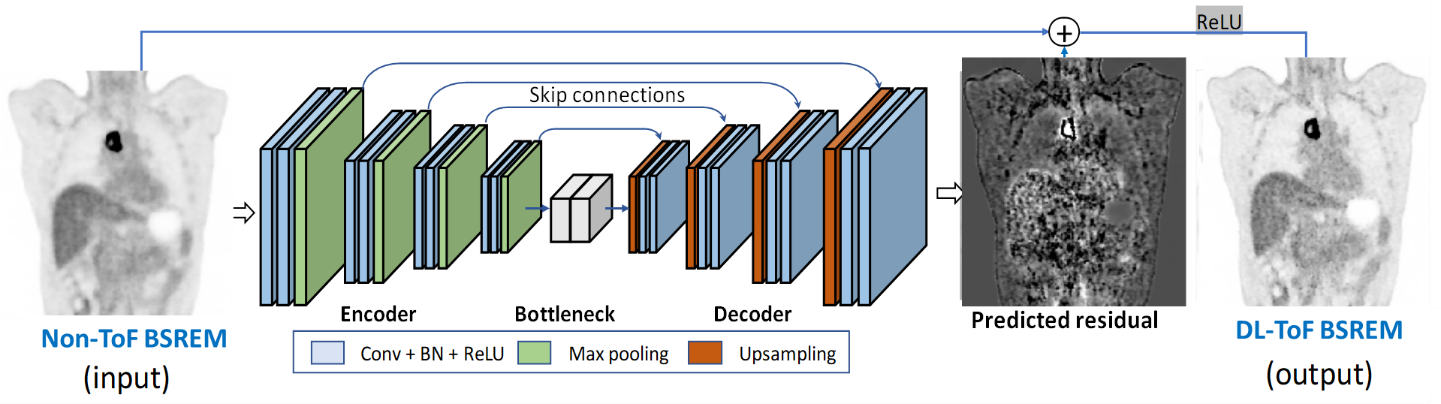


**Supplementary Figure 1.** The schematic architecture of the DL-ToF network based on a residual U-Net. The number of kernels is in the encoder 64, 128, 512, 1024.

**Supplementary Figure 2.** DL-ToF enhancement of a representative test subject with a BMI of 34.0 kg/m^2^ with an injected activity of 521 MBq scanned on GE Discovery MI (5-ring) PET/CT scanner (slice thickness 2.8 mm). Arrows point to lesions with lower detectability in non-ToF BSREM. Display window: 0-5 SUV. Top images show coronal slices, bottom image maximum intensity projections.

**Supplementary Figure 3.** DL-ToF enhancement of a representative test subject with a BMI of 30.8 kg/m^2^ with an injected activity of 479 MBq scanned on GE Discovery MI (5-ring) PET/CT scanner (slice thickness 2.8 mm). Arrows point to lesions with lower detectability in non-ToF BSREM. Display window: 0-5 SUV. Top images show coronal slices, bottom image maximum intensity projections.

**Supplementary Figure 4.** DL-ToF enhancement of a representative test subject with a BMI of 39.2 kg/m^2^ with an injected activity of 543 MBq scanned on GE Discovery MI (5-ring) PET/CT scanner (slice thickness 2.8 mm). Arrows point to lesions with lower detectability in non-ToF BSREM. Display window: 0-5 SUV. Top images show coronal slices, bottom image maximum intensity projections.

**Supplementary Figure 5** DL-ToF enhancement of a representative test subject with a BMI of 41.2 kg/m^2^ with an injected activity of 525 MBq scanned on GE Discovery MI (5-ring) PET/CT scanner (slice thickness 2.8 mm). Arrows point to lesions with lower detectability in non-ToF BSREM as well as the SUV_max_ of an example lesion. Display window: 0-5 SUV.

**Supplementary Figure 6.** DL-ToF enhancement of a representative test subject with a BMI of 28.6 kg/m^2^ an injected activity of 288 MBq scanned on a GE Discovery IQ non-ToF PET/CT scanner (slice thickness 3.8 mm). Display window: 0-5 SUV.

**Supplementary Figure 7.** DL-ToF enhancement of a representative test subject with a BMI of 38.2 kg/m^2^ with an injected activity of 218 MBq scanned on a GE Discovery IQ non-ToF PET/CT scanner (slice thickness 3.8 mm). Display window: 0-5 SUV.

**Supplementary Figure 8.** DL-ToF enhancement of a representative test subject with a BMI of 27.0 kg/m^2^ with an injected activity of 292 MBq scanned on a GE Discovery IQ non-ToF PET/CT scanner (slice thickness 3.8 mm). Display window: 0-5 SUV.


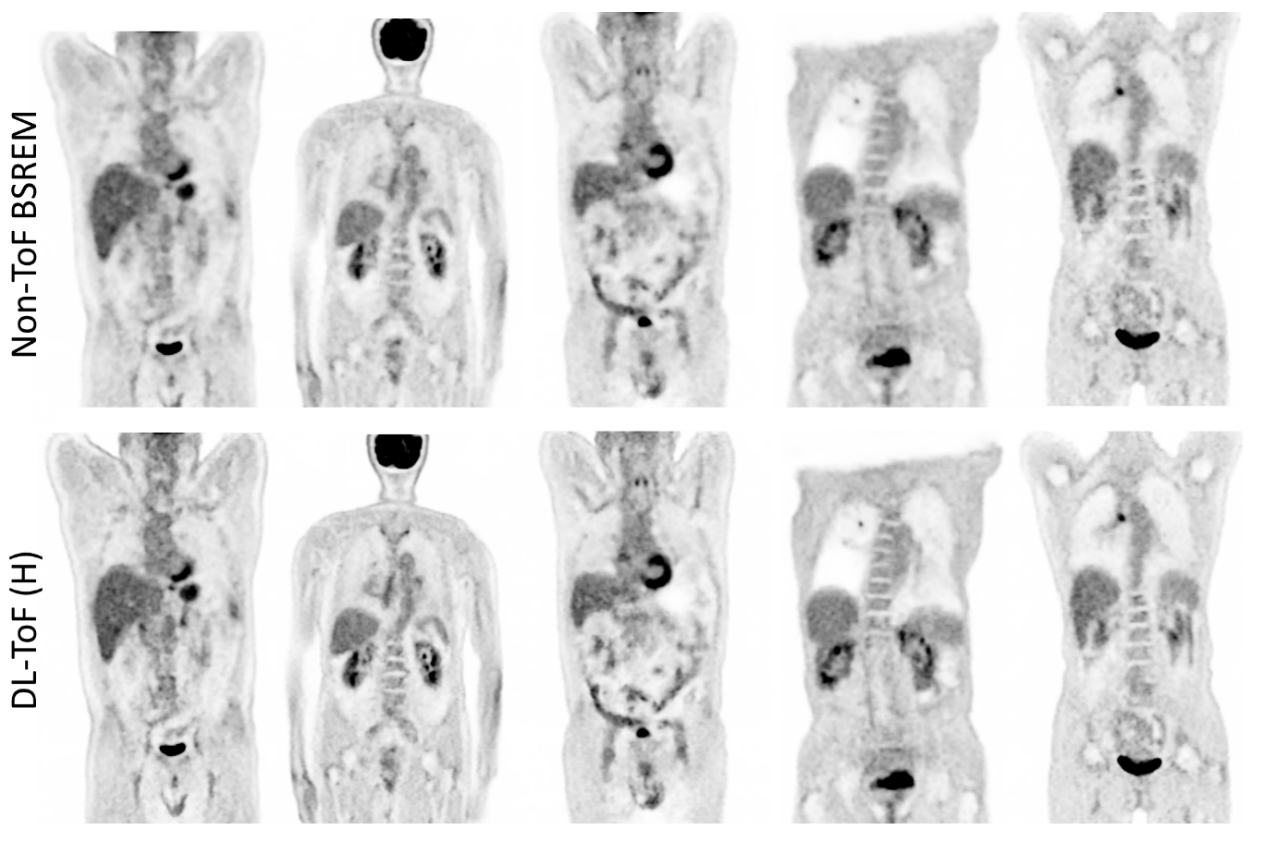


**Supplementary Figure 9.** DL-ToF enhancement of five representative test subjects scanned on a GE Discovery IQ non-ToF PET/CT scanner (slice thickness 3.8 mm). Display window: 0-5 SUV.
